# Linkages Between Trace Elements and Bacterial Communities in Glacial Freshwater Systems of Zhongar Alatau National Park, Kazakhstan

**DOI:** 10.1007/s00248-025-02674-2

**Published:** 2026-01-27

**Authors:** Lenka Pániková, Katarína Ondreičková, Patrik Pánik, Marián Janiga, Berikzhan Oxikbayev

**Affiliations:** 1 1Institute of High Mountain Biology, University of Žilina, Tatranská Javorina 7, 059 56 Žilina, Slovakia; 2 2National Agricultural and Food Centre, Research Institute of Plant Production, Bratislavská Cesta 122, 921 68 Piešt’any, Slovakia; 3 3Zhetysu University Named After Ilyas Zhansugurov, Zhansugurov St. 187 A, 040009 Taldykorgan, Kazakhstan

**Keywords:** Freshwater microbiome, High-mountain ecosystems, Metal–microbe interactions, ARISA community profiling, Meltwater-driven gradients

## Abstract

Glacial ecosystems of Central Asia represent extreme environments where microbial communities are shaped by both physicochemical conditions and hydrological dynamics. In this study, we analysed 21 surface and meltwater samples collected in September 2023 from a lake, river, glacier, glacial river, and sedimentary lake in the Zhongar Alatau National Park (Kazakhstan, 1 040–3 360 m a.s.l.). Bacterial community structure was assessed using ARISA profiling, while spectrometric methods determined concentrations of chemical elements. Alpha diversity indices revealed the highest richness and diversity in lake and sedimentary lake samples, moderate diversity in river samples, and the lowest values in glacier samples. The glacial river samples showed the strongest variability among the samples. Unique operational taxonomic units (OTUs) were most abundant in the lake, but the glacier exhibited the highest relative proportion of habitat-specific OTUs. Principal component analysis revealed that DNA yield, along with heavy metals and other elements (Rb, Fe, Mn, K, Ba), covaried along the major axes, primarily reflecting differences driven by habitat. Overall, our results demonstrate that glacial valley habitats host distinct bacterial assemblages and that the chemical environment is consistent with the observed spatial structuring of microbial communities. These findings highlight the vulnerability and sensitivity of mountain freshwater ecosystems to glacier retreat and associated changes in water chemistry.

## Introduction

Glaciers play a key role in the hydrological cycle of Central Asia [1, 2], acting as major freshwater reservoirs that regulate river discharge and sustain downstream ecosystems, especially during dry seasons [3, 4]. With globally increasing temperatures, glaciers around the world are retreating and losing volume [5, 6]. They act not only as reservoirs of freshwater but also as long-term sinks for atmospheric contaminants, such as heavy metals and persistent organic pollutants, which were deposited onto glacier surfaces in the past and continue to accumulate from ongoing atmospheric deposition [7]. During glacier melting, these legacy pollutants can be remobilized and released into downstream aquatic ecosystems [7]. The Zhongar Alatau is a Central Asian ridge of high mountains occurring between the Altai and the Tangshan. Arid deserts and lowlands are found along the border of these mountains. The frequency of dust storms directed into parts of the Zhongar Mountains has increased over the past few decades [8].

The shrinking of the Zhongar Alatau has the highest rate compared to other glaciated regions of the Central Asian mountains, including the Altai, Pamir, and even the Tien Shan [9]. Meltwater from these glaciers has distinctive physical, chemical and biological aspects that affect freshwater ecosystems [10]. Glaciers represent unique and dynamic ecosystems [11–13], and research on glacial microbiology has primarily focused on the supraglacial environment, largely due to its role in influencing the albedo of glaciers and ice sheets [14–19]. Even mountain glaciers, which have a small percentage of catchment area, can affect surrounding aquatic ecosystems [20, 21]. Turbidity in glacial meltwater stems from erosive activity of the glacier on the underlying bedrock [22]. Continued retreat often leads to the exposure of local basal depressions where proglacial lakes form [23]. Alpine lakes in high mountains and similar lakes in polar regions are inhabited by benthic microorganisms [24, 25]. These microbial communities consist of a unique diversity of bacteria, archaea, and microeukaryotes that drive key biogeochemical processes, such as organic matter decomposition, nutrient remineralization, and carbon and nitrogen cycling [24, 26, 27]. Nevertheless, their ecology, biodiversity and their linkage to chemical elements contained in sediments are a big unknown [28, 29]. Supraglacial ecosystems are characterized by low temperature, intense solar radiation, oligotrophic conditions, and high mineral content [30–33] suggesting that chemolithotrophic organisms could play an essential role in subglacial ecosystems [34, 35].

Contamination of glaciers has been assessed by comparing trace element concentrations with natural background levels derived from upper continental crust compositions and with data from other mountain and polar regions, including the Alps, Arctic, and Tibetan Plateau [36]. The origin of these elements can be attributed to transboundary atmospheric deposition, local dust inputs, and bedrock weathering processes [8, 37, 38]. Among redox-active metals, manganese and iron play a particularly important role in glacial and freshwater biogeochemistry. Manganese oxides act as strong sorbents of heavy metals and nutrients, forming natural sinks for contaminants [39], and their oxidation can be catalyzed by diverse bacteria and fungi [40], linking manganese cycling with microbial metabolism. Iron exhibits similar redox dynamics and serves as an essential micronutrient for nearly all living organisms, influencing enzymatic activity, photosynthesis, and overall redox balance [41, 42]. Through prolonged interaction with bedrock and glacial weathering, soluble bioactive forms of both iron and manganese accumulate in glacial basins and can be transported downstream with meltwater, affecting nutrient availability and microbial productivity in lakes and rivers [43, 44]. In addition, elements such as rubidium (Rb), potassium (K), and barium (Ba) are released during mineral weathering and enter aquatic systems primarily in dissolved form [45, 46]. Collectively, these geochemical processes shape the elemental composition of glacial meltwaters and contribute to the structure and functioning of microbial communities in glacier-fed ecosystems [12, 47].

The complex interactions between glacial hydrology, microbial communities, and geochemical processes are strongly influenced by ongoing climate warming [13, 48]. Although the accelerating retreat of glaciers and the transformation of surrounding environments are well documented, the microbial responses to these changes remain less understood [49]. This study, therefore, aimed to characterise the relationships between the chemical composition of glacial valley waters and the structure of bacterial communities in different aquatic habitats of the Zhongar Alatau Mountains. Specifically, we compared bacterial diversity and community composition among habitats directly influenced by glacier meltwater (glacier, glacial river, sedimentary lake) and those less affected (lake, river). By linking microbial and geochemical patterns across these interconnected environments, this research provides new insights into the ecological functioning and vulnerability of high-mountain freshwater ecosystems in Central Asia.

## Material and methods

### Study area

**Fig. 1:**
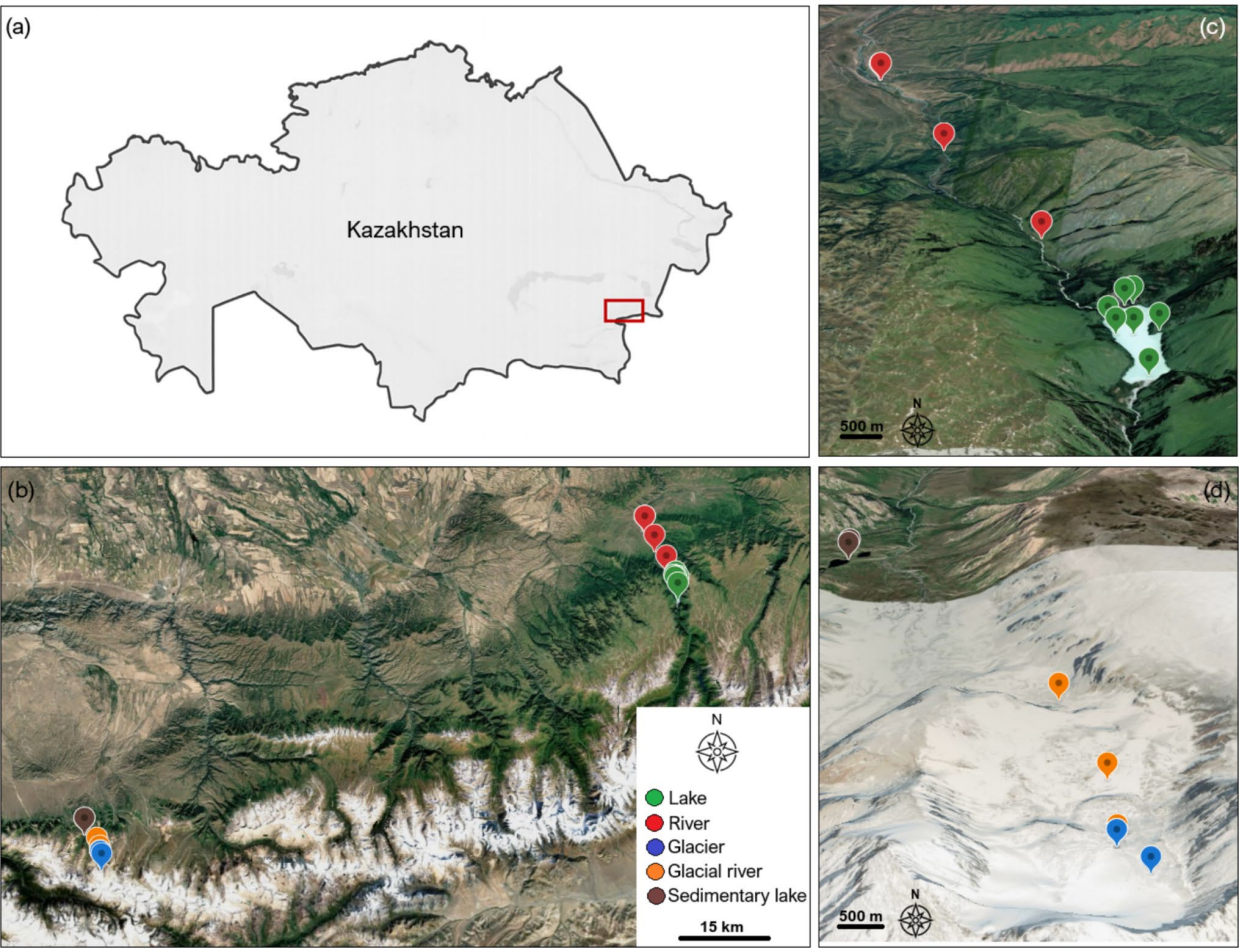
Location of sampling sites in the Zhongar Alatau Mountains, southeastern Kazakhstan. Sampled habitats cover an altitudinal gradient from 1 040 to 3 360 m a.s.l. (**a**) Geographic position of the study area within Kazakhstan (red rectangle). (**b**) Overview map showing two main sampling valleys within the Zhongar Alatau range, with all sampling sites classified by habitat type. (**c**) Detail of the northern valley containing lake (green) and river (red) habitats. (**d**) Detail of the southern glacial valley with glacier (blue), glacial river (orange), and sedimentary lake (brown) habitats. The map base was generated using Google Earth 10.91.0.1 (^©^Google LLC, 2025)

The research was conducted in the Zhongar Alatau National Park, situated in the Almaty Region of southeastern Kazakhstan near the border with China. The park, established in 2010, covers approximately 356 000 ha and encompasses diverse mountain landscapes of the Dzungarian (Zhetysu) Alatau Range [50, 51]. Sampling sites were distributed between 1 040 and 3 360 m a.s.l., representing a gradient from lowland river to high-altitude glacial and sedimentary lake (Tab. 1, Fig. 1). The region of Zhongar Alatau National Park is characterized by a continental mountain climate, with large diurnal and seasonal temperature fluctuations and uneven precipitation distribution throughout the year. Mean annual precipitation ranges from 300–400 mm, reaching up to 1 300–1 500 mm on the western slopes, with most precipitation falling as snow at higher altitudes. Average summer temperatures vary between + 10 and + 15 °C in the mountains and can exceed + 25 °C in the foothills, while winter temperatures frequently drop below −15 °C [50].

**Tab 1:** Geographic and environmental characteristics of the sampling sites in the Zhongar Alatau Mountains (Kazakhstan). The table lists the habitat type, geographic coordinates, sampling date, and altitude for all 21 surface water samples collected in September 2023. Lake (L1-L7) and river (R1-R4) samples were taken from the Zhasylkol area (northern valley), whereas glacier (G1-G5) glacial river (GR1-GR3) samples originated from the upper part of Ushkol area (southern valley). Sedimentary lake samples (SL1-SL2) were collected from the Ushkol moraine lakes located directly below the glacier

Sample	Habitat	Latitude	Longitude	Sampling date	Altitude (m a.s.l.)
L1	Lake	45.3876525	80.58221917	Sep 14, 2023	1 650
L2	Lake	45.3780775	80.5810825	Sep 14, 2023	1 650
L3	Lake	45.38707694	80.57798028	Sep 14, 2023	1 650
L4	Lake	45.38961972	80.57352444	Sep 16, 2023	1 650
L5	Lake	45.38710528	80.57501194	Sep 16, 2023	1 650
L6	Lake	45.39390722	80.5759525	Sep 14, 2023	1 650
L7	Lake	45.39441417	80.57734944	Sep 14, 2023	1 650
R1	River	45.41782667	80.55845333	Sep 16, 2023	1 280
R2	River	45.44842917	80.53421222	Sep 16, 2023	1 210
R3	River	45.47602306	80.51447889	Sep 17, 2023	1 040
R4	River	45.4761675	80.51466889	Sep 17, 2023	1 040
G1	Glacier	44.98903611	79.39339639	Sep 21, 2023	3 280
G2	Glacier	44.98903611	79.39339639	Sep 21, 2023	3 280
G3	Glacier	44.98903611	79.39339639	Sep 21, 2023	3 280
G4	Glacier	44.98903611	79.39339639	Sep 21, 2023	3 280
G5	Glacier	44.9856025	79.39630222	Sep 21, 2023	3 360
GR1	Glacial river	44.98967806	79.39333222	Sep 21, 2023	3 265
GR2	Glacial river	44.99651556	79.39159306	Sep 22, 2023	3 200
GR3	Glacial river	45.00787806	79.38572972	Sep 22, 2023	3 015
SL1	Sedimentary lake	45.035845	79.35863583	Sep 22, 2023	2 535
SL2	Sedimentary lake	45.03629583	79.35863833	Sep 22, 2023	2 530

Sampling was conducted in two parts of the Zhongar Alatau range: (i) Zhasylkol area (northern valley, Fig. 1c) included a large oligotrophic mountain lake Zhasylkol (L1-L7; 1650 m a.s.l.) and its associated river sections (R1-R4; 1 040–1 280 m a.s.l.). The lake is situated at the foot of the northern slope of the range and is primarily fed by snowmelt and surface runoff. (ii) Ushkol area (southern valley, Fig. 1d) comprised glacier (G1-G5; 3 280–3 360 m a.s.l.), glacial river (GR1-GR3; 3 015–3 265 m a.s.l.), and sedimentary lake (SL1-SL2; 2 530–2 535 m a.s.l.) habitats. The sedimentary lakes, locally known as Ushkol lakes, are located directly below the glacier terminus and are connected to meltwater channels draining the glacier.

### Sampling

Water samples (up to 2 L per site) were collected into sterile polypropylene bottles. Bottles were opened only immediately before immersion (10–20 cm below the surface) and handled with sterile gloves, which were changed at each site. To prevent cross-contamination, single-use sterile consumables were prioritised; where reusable metal tools were required, they were decontaminated between sites using 10% sodium hypochlorite (1–2 min), rinsed with sterile water, wiped with 70% ethanol and air-dried. Samples were transported to the laboratory in cooled insulated containers (4 °C) within 24 h of collection. Upon arrival, each water sample was divided into two 1 L subsamples: one for elemental analysis and one for molecular analysis, which were vacuum filtered through sterile 0.22 µm membrane filters. For DNA analysis, the filter membrane was stored at −20 °C until the extraction of DNA.

### Laboratory analysis

For the determination of elemental composition, up to 1 L of water subsamples were filtered through disposable filtration units equipped with 0.22 µm membrane filters. Filtration was assisted by a vacuum pump (model N86KN.18, KNF, Germany), maintaining a thin water layer (1 cm) above the membrane to prevent desiccation. After filtration, the membranes were air-dried, and the retained material was used for elemental analysis. Concentrations of selected chemical elements (K, Ba, Rb, Fe, Mn, Mo, Ni, Cr, and Hg) were determined using an ED-XRF spectrometer DELTA Professional with XRF WorkStation (Olympus, Innov-X, USA). Element concentrations were quantified based on the intensity of characteristic X-ray emission lines. Instrument calibration and analytical accuracy were verified using a certified reference material — bovine liver standard (NCS ZC 7001, CHNACIS, China). Mercury was analysed separately using a Milestone DMA-80 evo instrument, based on thermal decomposition, gold-amalgamation, and atomic absorption spectrometry.

### Molecular analysis of bacterial communities

DNA extraction was performed from the frozen filters obtained after filtration of the second water subsamples designated for molecular analyses. The actual filtered volume varied depending on turbidity: in clear lake samples, approximately 1 L could be processed, whereas in highly turbid habitats, such as glacial rivers or sedimentary lakes, filters clogged earlier, and only 700–800 mL could typically be filtered. Entire filters were used for DNA extraction using the DNeasy Power Water Kit (Qiagen), regardless of sediment content, using consistent extraction protocols and normalization of elution volumes (50 μl) across all samples helped minimise technical bias. The purity of DNA was measured spectrophotometrically with the NanoDrop One Spectrophotometer (Thermo Scientific Inc., Wilmington, USA). Although sediment mass per sample was not directly measured, dsDNA (double-stranded DNA) concentration was fluorescently quantified using a Qubit™ Flex fluorometer (Invitrogen) with Qubit dsDNA HS (High Sensitivity) Assay Kit, and samples were diluted to the same final concentration (20 ng/μl), and stored at −20 °C. Automated ribosomal intergenic spacer analysis (ARISA) was used for detection of genetic diversity of bacterial communities in the water samples. The ITSF/ITSReub [52] primer set with 6-FAM fluorescent dye on the 5´ end of the reverse primer was used for amplification of the 16S-23S rRNA intergenic transcribed spacer (ITS) region from the bacterial rRNA operon. DNA amplification of bacterial communities was carried out in 50 µL reaction mixture containing 1 × PCR buffer (Invitrogen, Thermo Fisher Scientific Inc., Waltham, USA), 1.5 mmol Mg^2+^, 0.25 μmol of both primers, 200 μmol of each dNTP (Invitrogen, Thermo Fisher Scientific Inc., Waltham, USA), 1 U Taq DNA polymerase (Invitrogen, Thermo Fisher Scientific Inc., Waltham, USA), and 1 μL (20 ng) of DNA extracted from the rhizosphere. The PCR was performed in a GeneAmp PCR System 9700 (Applied Biosystems, Thermo Fisher Scientific, Inc., USA) using the following conditions: initial heat denaturation at 94 °C for 3 min, followed by 35 cycles each consisting of a denaturation step at 94 °C for 45 s, annealing at 60 °C for 1 min, extension at 72 °C for 2 min and a final extension step at 72 °C for 10 min. PCR amplification was confirmed by horizontal electrophoresis on a 1% (w/v) agarose gel in 1 × TBE buffer pre-stained with 0.10 µL/mL of ethidium bromide and visualised using UV illumination. PCR products were precipitated with ethanol and dissolved in 10 µL of sterile water. One microliter of purified products was added to 9 µL formamide containing LIZ1200 size standard (Applied Biosystems, Thermo Fisher Scientific, Inc., USA), denatured at 95 °C for 3 min and separated by capillary electrophoresis using ABI 3100 Prism Avant (Applied Biosystems, Thermo Fisher Scientific, Inc., USA). Outputs from ARISA in the form of electropherograms were analysed by the Peak Scanner 2 (Applied Biosystems, Thermo Fisher Scientific Inc., Wilmington, USA), and OTUs (Operational Taxonomic Unit) in range 200–1000 bp were used for the evaluation. Only peaks above the threshold of 50 fluorescence units were considered.

### Statistics

Statistical analyses were performed using Statistica Ver. 12 software. We used PCA to determine the relationship between sites with elements and sites with bacteria.

### Molecular data statistics

ARISA profiles were used to define operational taxonomic units (OTUs) based on unique fragment lengths, with each distinct peak representing a putative genetically distinct bacterial population. To account for minor variability in fragment sizing and reduce artificial inflation of OTU richness, peaks within a ± 1 bp window were merged into a single OTU (“binning”), following the recommendations of Ramette [53]. For downstream diversity calculations, raw peak heights (measured as relative fluorescence units, RFU) were normalised within each sample. Specifically, the RFU of each OTU was divided by the total RFU of all peaks detected in that sample, yielding relative OTU abundances expressed as proportions summing to 1 (i.e., 100%). This normalisation ensured that diversity indices reflected community composition independently of absolute fluorescence intensity, which can vary due to technical factors such as PCR efficiency or DNA template concentration. Alpha diversity indices (Chao-1, Shannon, Simpson, Evenness) were then calculated from these normalised profiles. Chao-1 was based on the number of distinct OTUs (including singletons), while Shannon, Simpson, and Evenness indices incorporated the relative abundance information derived from normalised RFU values. Since ARISA does not provide taxonomic identification, these indices reflect fragment-defined OTU richness and evenness, rather than true species richness. Statistically significant differences among groups of samples were tested using ANOVA at the 95% confidence interval for the means, followed by post hoc LSD test using the software Statgraphics × 64 (Statpoint Technologies, Inc., Warrenton, USA). To illustrate OTU overlap across habitats, an UpSet plot was generated using the *UpSetR* package via Gehlenborg Lab’s online Shiny app (https://gehlenborglab.shinyapps.io/upsetr/) [54]. To further visualise the connectivity among samples, a network analysis was performed in PAST v4.17 [55] using Euclidean similarity matrices generated from normalised ARISA profiles. Networks were constructed at two similarity thresholds (50% and 75%) to explore patterns of shared OTUs within and among habitat types. Node size was scaled according to the number of OTUs in each sample, and edges were drawn when the similarity between two samples exceeded the given threshold. Also, bacterial communities in different samples were compared using fluorescence intensity values of individual OTUs. These data were subsequently used for principal component analysis (PCA) with a correlation matrix. PCA was also combined with alpha diversity indices from molecular data and with chemical element concentrations to construct integrative ordination plots. All alpha diversity indices and PCA analyses were performed using the PAST software version 4.17.

## Results

### Bacterial diversity

**Fig. 2:**
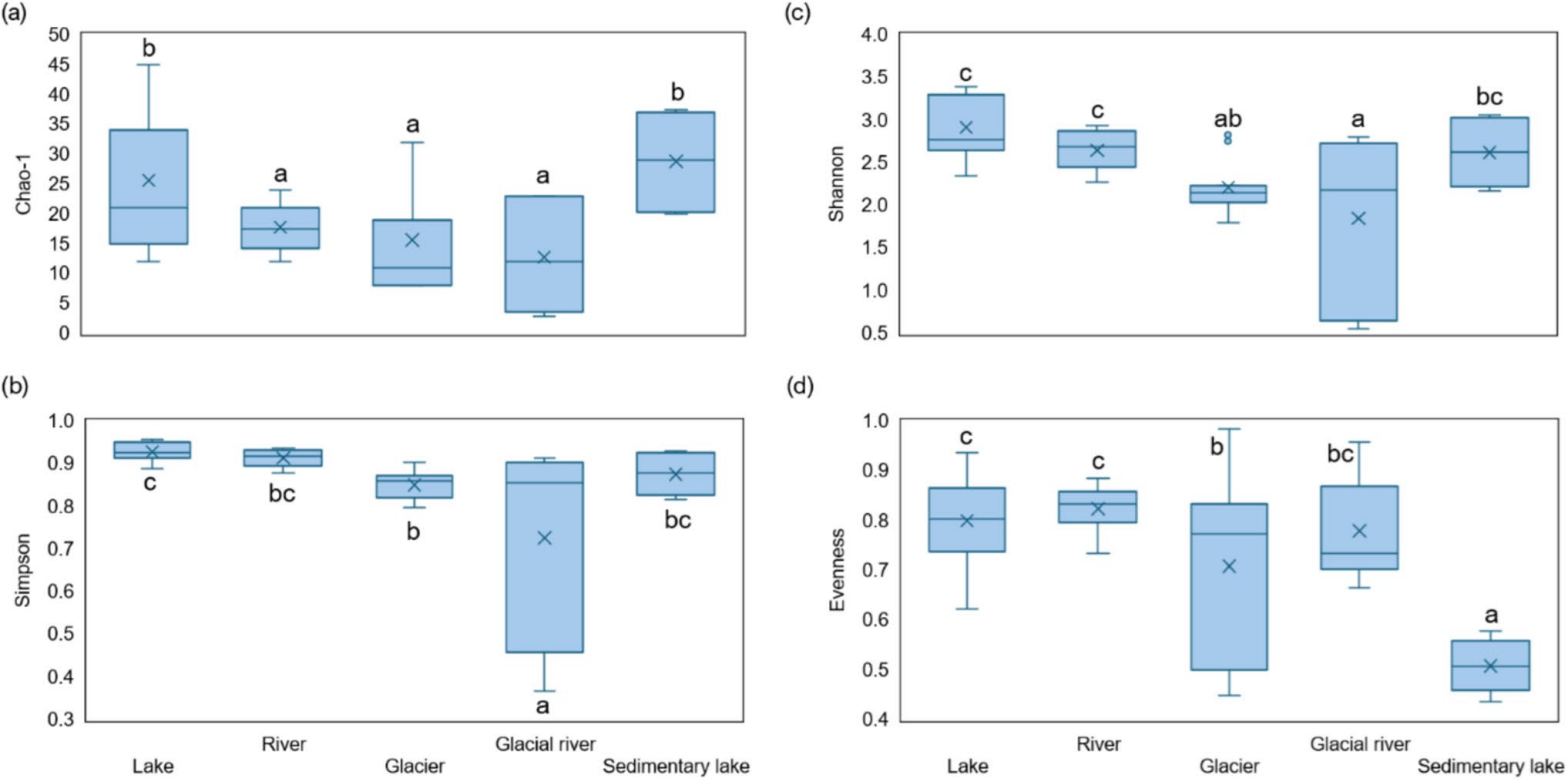
Alpha diversity indices (Chao-1, Simpson, Shannon, and Evenness) of bacterial communities detected in water samples. Different lowercase letters correspond to the statistically significant differences among aquatic habitats (LSD, P ≤ 0.05)

The alpha diversity indices (Fig. 2) indicate that lake exhibited the highest OTU richness (Chao-1) and the highest overall diversity (as measured by Shannon, Simpson, and Evenness indices). These indices suggest that bacterial communities within lake were well-distributed and diverse. River samples also showed high diversity (Shannon, Simpson, Evenness), comparable to that of lake, but their OTU richness (Chao-1) was moderate, suggesting a slightly lower number of species. In contrast, glacier samples demonstrated lower OTU richness (Chao-1), and diversity (Shannon, Simpson) compared to lake and river. The species evenness in glacier was medium, which implies that certain species/OTUs could dominate in the community. Samples from glacial river had the most variable values across nearly all indices (Chao-1, Shannon, Simpson), indicating low bacterial diversity in some samples and the dominance of a few OTUs. Meanwhile, sedimentary lake samples had high OTU richness (Chao-1) but exhibited low evenness (Evenness), suggesting that a limited number of species dominate these environments. The diversity values measured by Shannon and Simpson were medium in comparison to other aquatic habitats.

**Fig. 3:**
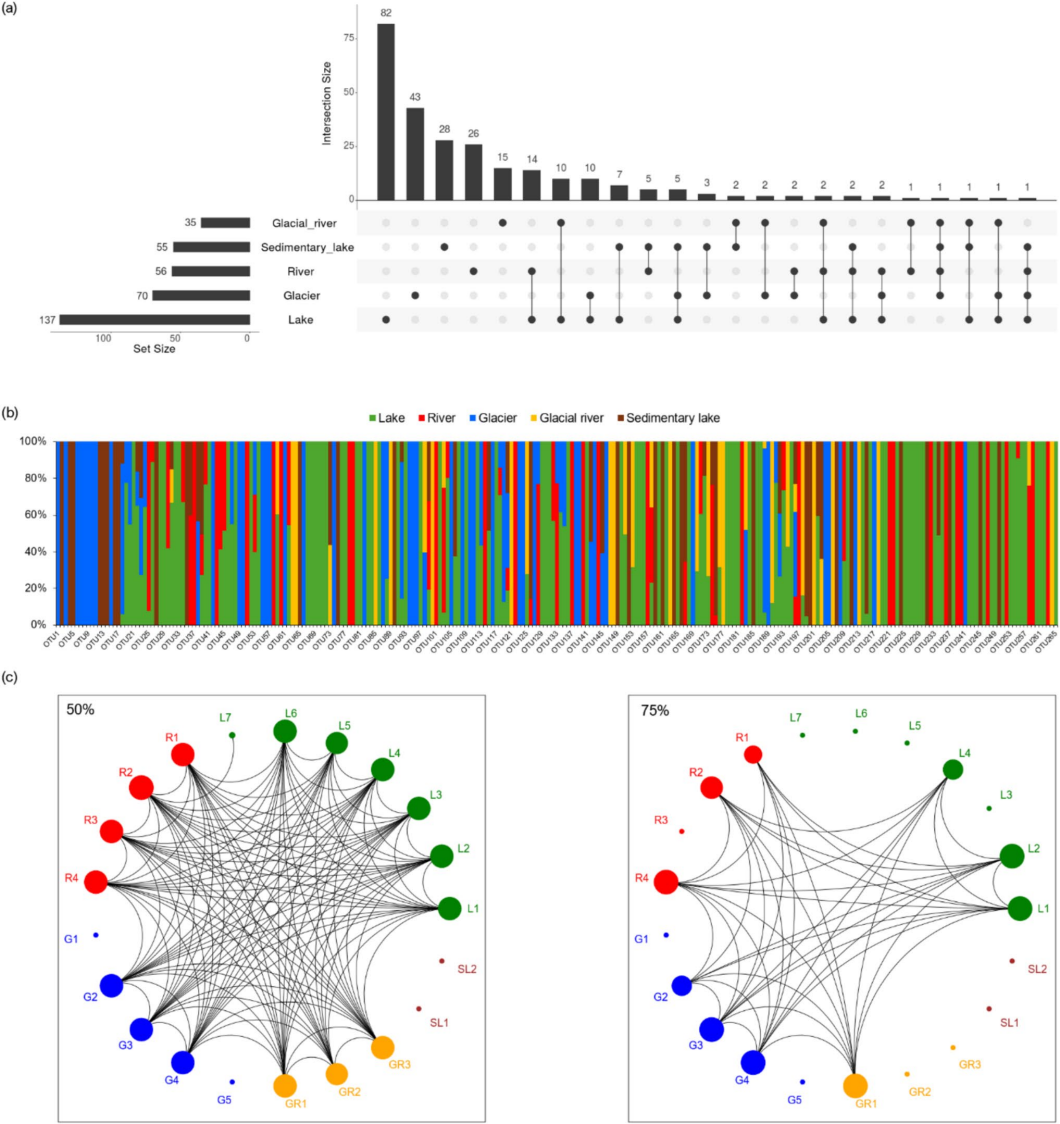
Bacterial community patterns across aquatic habitats based on ARISA profiles. (**a**) Upset plot displaying unique and shared bacterial OTUs across five aquatic habitats based on ARISA profiles. Vertical bars represent the number of OTUs detected in specific habitat combinations, while the connected dots below indicate the corresponding sets of habitats. Horizontal bars on the left display the total number of OTUs detected in each habitat. (**b**) Stacked bar chart showing the relative distribution of OTUs (n = 265) across habitats. Each bar represents one OTU, and colours indicate its relative abundance in different habitat types. (**c**) Network plots illustrating similarity relationships among samples at 50% (left) and 75% (right) Euclidean similarity thresholds. Nodes represent individual samples, and edges connect samples with similarity above the specified threshold. Clustering of nodes reflects community-level resemblance among habitats. Node size is scaled by the number of connections (degree), representing how strongly each sample is linked to other samples at the given similarity threshold

According to the Upset plot (Fig. 3a), the lake habitat contained the highest absolute number of unique OTUs, with 82 detected, representing 59.9% of all OTUs found in lakes. The glacier habitat followed in absolute numbers, with 43 unique OTUs; however, these accounted for the highest relative proportion, 61.4% of all glacier OTUs. Sedimentary lakes and rivers showed comparable levels of unique bacterial taxa, with 28 (50.9%) and 26 (46.4%), respectively. In contrast, the glacial river exhibited only 15 unique OTUs (42.9%), indicating the lowest degree of specialisation relative to the other ecosystems. In terms of shared bacterial taxa among different habitats, the glacial river shared few taxa with other environments, which could reflect its extreme conditions. Conversely, the most shared taxa were found between lake and river habitats (14 shared OTUs) and between lake and glacier habitats (10 shared OTUs). Notably, no bacterial OTUs were common across all habitats. The stacked bar chart (Fig. 3b) provides a complementary view of the relative distribution of all detected OTUs (n = 265) across habitats. Community-level relationships are illustrated by the network analysis (Fig. 3c). At the 50% similarity threshold, most samples were interconnected, forming a dense network indicative of shared community members across habitats. At the 75% similarity threshold, lake, river, and glacier samples formed distinct, internally cohesive clusters, whereas glacial river samples appeared more fragmented, and sedimentary lakes remained largely isolated.

### Bacterial diversity and chemical elements

**Fig. 4:**
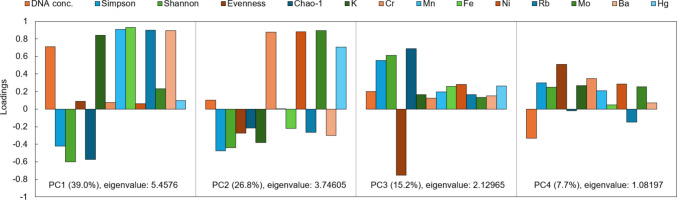
The component loadings for DNA concentration, indices of bacterial alpha diversity, and amounts of chemical elements from the first four significant principal components in PCA

Principal component analysis (PCA) was used to evaluate the relationships between bacterial alpha diversity, DNA concentration and chemical element content in water samples from different types of aquatic environments (lake, river, glacier, glacial river and sedimentary lake). The results showed that the first four principal components with eigenvalues higher than 1 were significant (Fig. 4). PC1 explained 39.0% of the variability and correlated with the increase in the concentrations of elements such as Rb, Fe, Mn, K and Ba, which had high positive loadings on PC1 (Rb: 0.8996, Fe: 0.93156, Mn: 0.90684, K: 0.84095, Ba: 0.89403). DNA concentration also had a high positive loading on PC1 (0.7098), clustering together with Rb, Fe, Mn, K, and Ba. This suggests that DNA yield and element concentrations covary across habitats, although the observed pattern most likely reflects broader habitat-driven differences rather than direct causality. Conversely, bacterial diversity indices (Chao-1, Shannon, Simpson) had negative loadings on PC1 (e.g. Simpson: −0.42315, Shannon: −0.59845, Chao-1: −0.57411), indicating that higher PC1 values were associated with lower bacterial diversity represented by alpha diversity indices. In other words, samples with high DNA concentrations and high levels of the above chemical elements had low bacterial diversity, and vice versa, samples with lower concentrations of DNA had higher bacterial diversity indices. Thus, the variability on the PC1 axis is substantially influenced by the concentration of DNA in nature and the ability to detect it quantitatively in the laboratory. However, it is noteworthy that the diversity of bacteria decreases in those samples where more Rb, Fe, Mn, K and Ba were present simultaneously. PC2 explained 26.8% of the variability and correlated with element concentrations such as Ni, Cr, Mo, and Hg, which had high positive loadings on PC2 (Cr: 0.87581, Ni: 0.88205, Mo: 0.89487, Hg: 0.705). Thus, PC2 is the main vector to characterize the co-occurrence of heavy metals (Cr, Ni, Mo, Hg), as their concentration is independent of the DNA concentration, but the bacterial diversity in these samples tends to decrease. This is a notable pattern indicating that samples with higher heavy metal concentrations tend to exhibit lower bacterial diversity. PC3 explained 15.2% of the variability in the data. Variables with high positive loadings on PC3 were the bacterial diversity indices Shannon (0.61263), Simpson (0.55548) and Chao-1 (0.69064). This suggests that PC3 revealed variability in the data, which was primarily associated with differences in bacterial diversity. Samples with high PC3 values were characterized by high bacterial diversity. Conversely, evenness had a high negative loading on PC3 (−0.75192), indicating that a high PC3 value was associated with low evenness of species representation. PC4 explained 7.7% of the variability in the data. Variables with higher positive loadings on PC4 were evenness (0.50763) and Cr (0.34986) indicating that PC4 primarily captured variation in the uniformity of OTU abundance distributions together with moderate differences in Cr concentrations. Samples with higher PC4 scores, therefore, exhibited more even bacterial communities and slightly elevated chromium levels. The DNA concentration showed a moderate negative loading (−0.33019), suggesting that samples with higher DNA yields tended to have lower PC4 values.

**Fig. 5:**
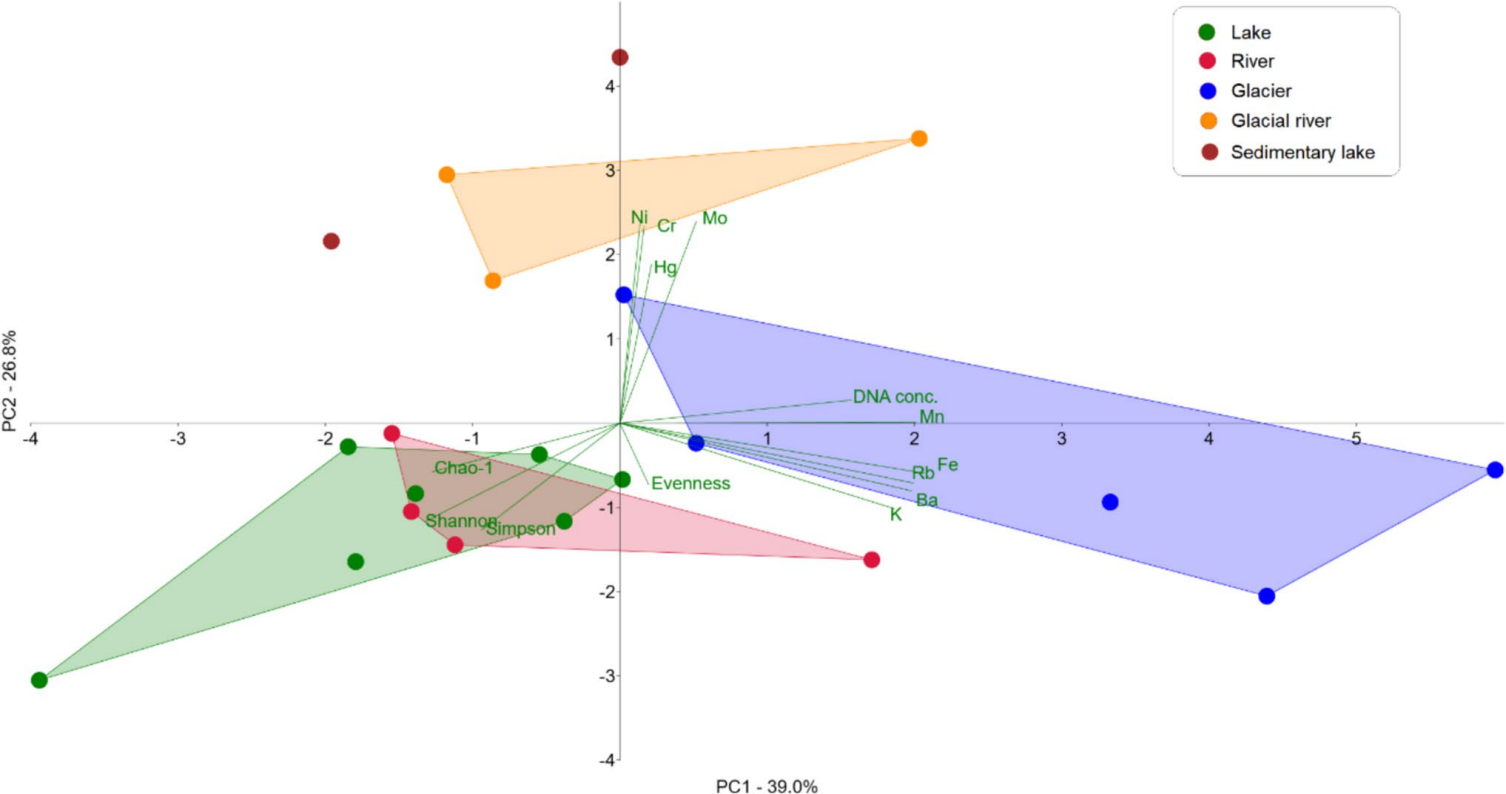
PCA biplot created from DNA concentration, bacterial community alpha diversity indices, and chemical element amounts

The PCA visualization (Fig. 5) showed the clustering of samples according to their origin at the mentioned first two components. Lake samples (green dots) were in the lower left quadrant, with their distribution influenced by higher diversity values (Shannon index, Simpson index and Chao-1). River samples (red dots) were close to lake samples, indicating a similarity in bacterial communities in these indices. Glacier samples (blue dots) were clustered on the right side of the graph, indicating different chemical properties, especially higher Fe, Mn, Rb, Ba, and K, as well as being associated with higher DNA concentration. Glacial river samples (orange dots) showed a variable distribution, with their chemical characteristics influenced by the input of elements such as Cr, Ni, Mo and Hg. Sedimentary lakes (brown dots) were distinguished by high values on PC2, indicating an increased content of metals such as Hg, Cr, Mo and Ni. These samples also showed slightly lower values of DNA concentration than those in the glacier, indicating that DNA extraction in environments rich in particulates did not experience significant inhibition. Additionally, the relatively high alpha diversity indices observed in sedimentary lake samples suggest that any potential PCR inhibition present did not significantly affect downstream amplification.

### Environmental similarity—chemistry and bacterial communities

**Fig. 6:**
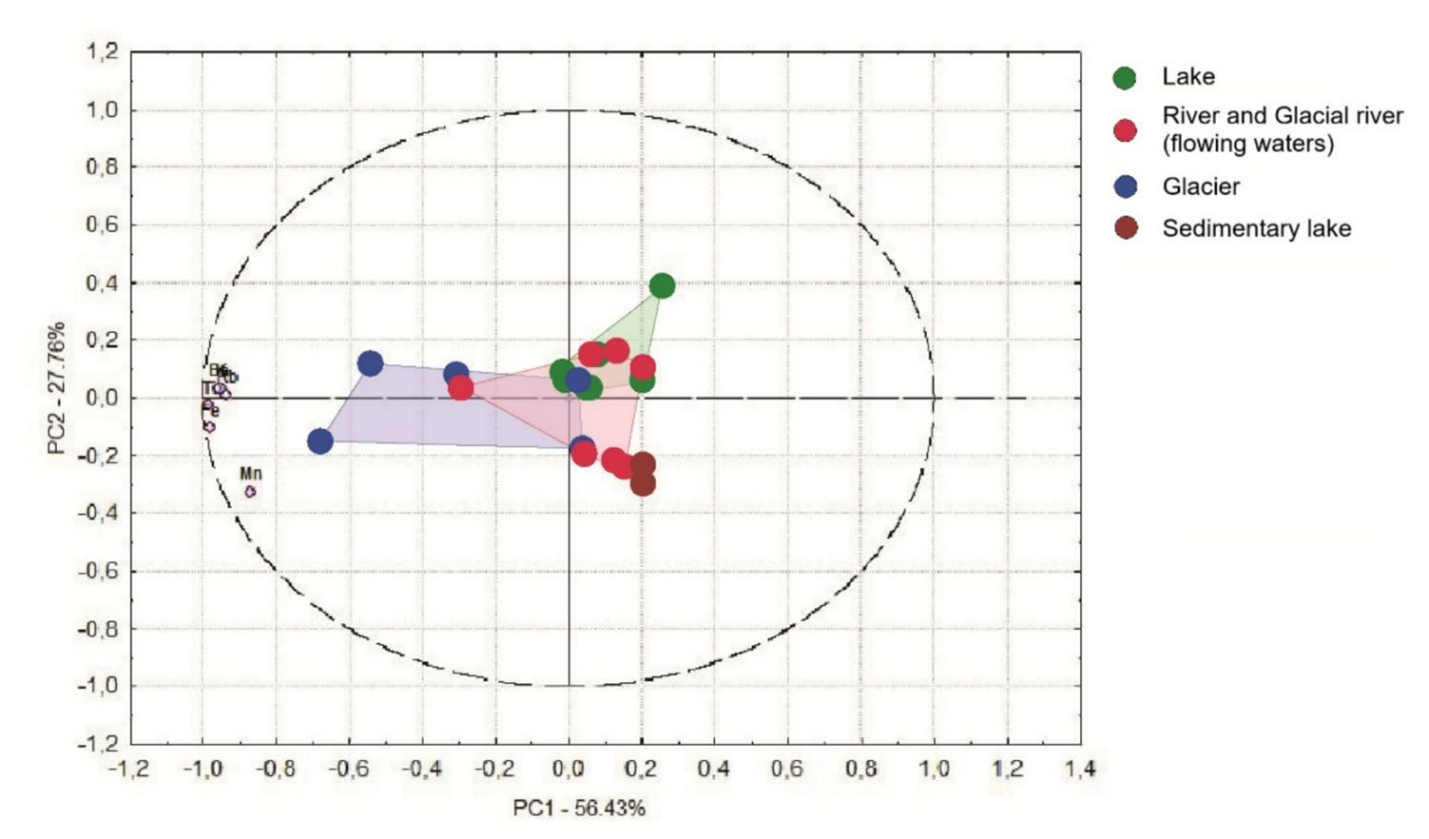
PCA biplot created from chemical element concentrations across sampling sites in the Zhongar Alatau Mountains. The ellipse represents the 95% confidence interval around the group centroid

**Fig. 7:**
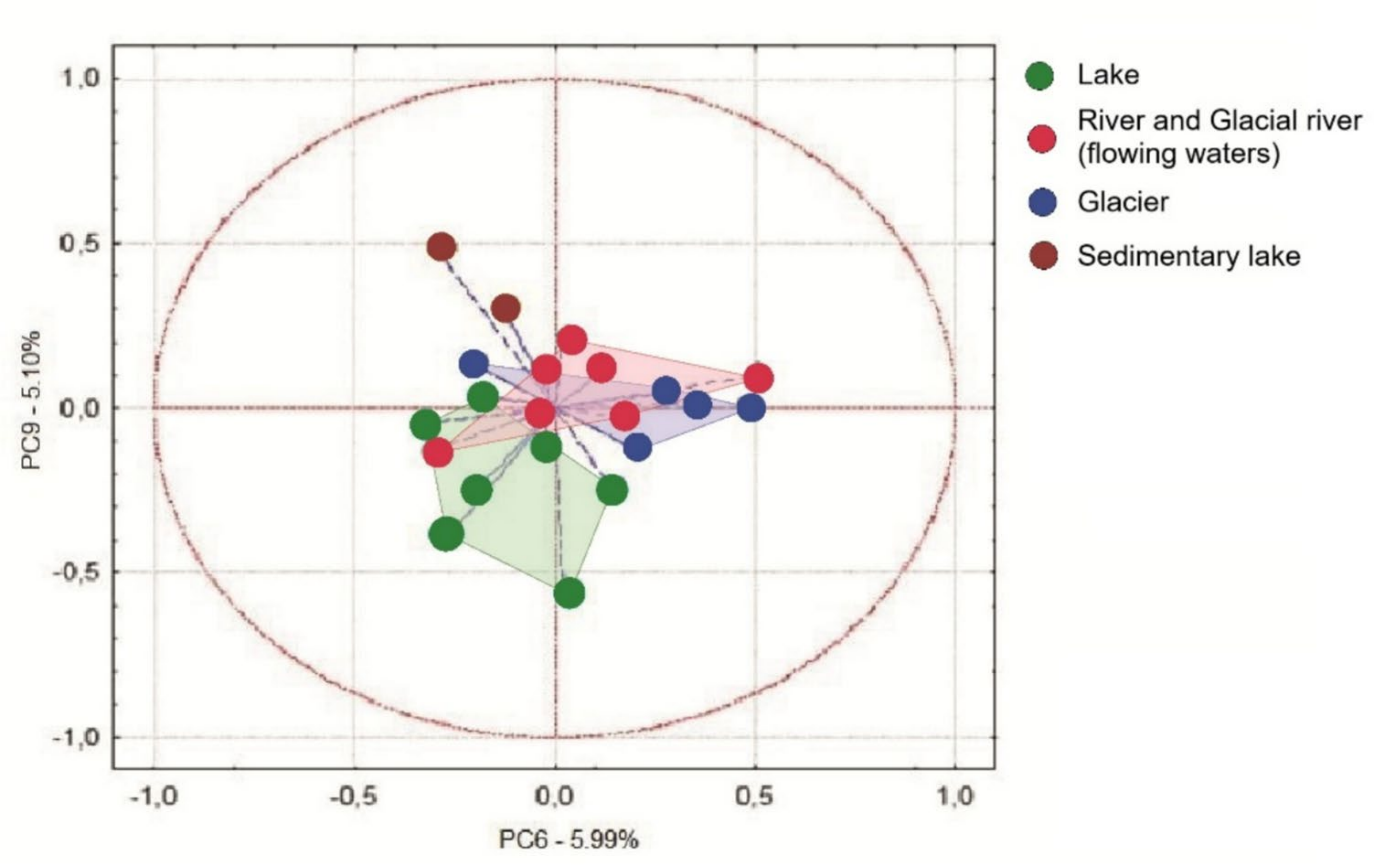
PCA plot of bacterial ARISA profiles across sampling sites in the Zhongar Alatau Mountains. The ellipse represents the 95% confidence interval around the group centroid. Habitat stratification parallels the chemical PCA (Fig. 6). The most comparable environmental patterns were captured by principal components 6 and 9, indicating a link between bacterial community structure and water chemistry shaped by Mn, Rb, K, Ba and Fe. Glacier communities differed from those of other aquatic ecosystems (PC6 – x-axis), while sedimentary lake samples were distinctly separated along PC9 (y-axis)

For visualization and comparative purposes, river and glacial river samples were merged into a single category representing flowing waters, reflecting their shared role as transport systems linking glacial meltwaters with downstream aquatic environments. The PCA constructed from chemical element concentration data alone indicates the occurrence of the two most important trends in glacial valleys. These trends may be independent and could reflect different underlying processes. The most substantial observation (PC1, 56.4% of data variance) is the synergistic accumulation of Mn, Ca, Rb, K, Ba and Fe, particularly evident in samples collected from the glacier (Fig. 6). The second observation reflects considerable accumulation of manganese (PC2, 27.8%), manifested mainly in a high-mountain sedimentary lake directly below the glacier. The environmental structure revealed by the first two axes of the chemical PCA has an analogue in the distribution of bacterial communities (Fig. 7). Similar environmental patterns were also captured by components PC6 (x-axis, Fig. 7) and PC9 (y-axis). Along the x-axis, glacier samples differed notably from sedimentary lake samples, whereas along the y-axis, sedimentary lake samples were distinctly separated from lake samples. We observed that the dominant chemical environment of glacial valleys similarly varied with the spatial patterns of bacterial community structure. The first two principal components of the chemical dataset explained 84.2% of the total variance (PC1 = 56.4%, PC2 = 27.8%), indicating strong environmental structuring among habitats (Fig. 6). Similarly, PCA based on ARISA profiles showed analogous grouping patterns of samples (Fig. 7), suggesting that microbial community composition could reflect the underlying environmental gradients.

## Discussion

Large amounts of trace elements entering the atmosphere from anthropogenic emissions can have an adverse effect on the environment. In high mountain areas, glaciers play an important role for pollution accumulation. The southeastern mountainous territory of Kazakhstan and its glaciers is influenced by Kazakhstan's economy, based on the extraction of mineral resources such as uranium, chromium, zinc, and manganese. The country's strong industrial development in mining, smelting and energy, leads to relatively large emissions of air pollutants [56–58] also affecting the Zhongar Alatau Mountains.

Analysis of bacterial diversity in different aquatic habitats showed significant differences in OTU richness, evenness and overall diversity. Although ARISA provides high-resolution fingerprinting of microbial communities, its fragment-based nature means that inferred OTUs are not taxonomically resolved. Therefore, we considered diversity interpretations in the context of community-level genetic variability, not as precise taxonomic richness. Lake samples showed the highest bacterial species richness and are probably home to more stable and complex microbial communities compared to other habitats. Sedimentary lake just below the glacier was also characterized by high OTU richness and diversity of bacteria but had very low community equilibrium. Low evenness may indicate the dominance of certain bacterial species. River samples showed similar diversity values to lakes but with lower species richness. Even though lakes and rivers within the study area may be connected through surface or subsurface flow, the number of shared OTUs between these habitats was limited. This may be attributed to selective environmental pressures, differing sediment loads, glacial influence, and local biotic interactions. It also reflects the fragment-based resolution of ARISA, which may not detect subtle taxonomic overlaps if fragment sizes coalesce. Conversely, glacier and glacial river showed overall lower diversity. The subglacial environment is consistently dark [59, 60] making it fundamentally different from the glacial meltwater environment [61]. Nevertheless, the subglacial zone can still serve as a source of microbial cells that are transported into meltwaters, where they can be detected by DNA-based methods even if they are not thriving or metabolically active in situ [62, 63]. Bacteria in meltwater can originate from the glacier or the surrounding environment, being transported by wind or snow from nearby aquatic and terrestrial sources bound to dust or organic particles [64–66]. The diverse bacterial communities in glacial lakes and the pattern of their distribution play a key ecological role in biogeochemical cycling and mineral nutrient cycling [11, 47]. Seasonal changes, including air temperature, nutrient concentrations, and solar radiation, influence microbial community structure by favouring only those species that can cope with environmental stresses [67]. The study of Bradley et al. [68] highlights that glacier and ice sheet surfaces are home to diverse microbial communities whose activity directly influences biogeochemical cycles and ice melting. A crucial aspect of their survival is dormancy, allowing them to rapidly reactivate upon thawing, showcasing their adaptability to extreme and dynamic conditions. This research emphasizes that these surface environments and their microbial inhabitants are highly vulnerable to climate change that may lead to shifts in those communities.

Sedimentary lake samples, which had high alpha diversity of bacterial communities (Fig. 2), are essential for understanding biodiversity responses to glacial retreat. Distance between glacial lakes and the glacier appears to be key as a driver of microbial diversity in lakes [67]. Analysis of common and unique bacterial communities revealed that the highest number of unique bacterial groups was found in lake environments, whereas the number of unique bacterial groups was very low in glacial river. This low diversity and species specificity may be attributed to significant physicochemical stressors such as low temperatures or high influx of adjacent glacial debris from melting glaciers [69]. Glacier-derived bacteria can contribute significantly (40–96%) to the stream microbial communities of a glacial lake, but their contribution decreases with increasing distance from the glacier terminus [70]. Liu et al*.* [71] found that the lake closest to the retreating alpine glacier had higher diversity than other lakes. Melted glacial water contains particles that cause high turbidity, reducing the amount of photosynthetically active radiation [25]. Further, containing high concentrations of nutrients, lakes fed by this water have the potential to be unique ecosystems and hotspots of nutrient cycling [72]. Lake sediments offer a solid, nutrient-enriched surface that is readily colonized by microbes, while water has the opposite effect, dissolving potentially toxic elements in the water and further reducing microbial diversity [73].

Guo et al*.* [74] discuss that increased glacier melting due to global warming expands lake areas, and the massive influx of glacial meltwater significantly affects microbial assemblages. This occurs through direct export of microorganisms from glaciers and indirectly by altering abiotic factors like turbidity, which limits light penetration and impacts primary producers. As glaciers retreat, and their meltwater influence diminishes, the microbial sources for these lakes are expected to shift towards non-glacial streams, leading to major changes in the physicochemical characteristics and microbial communities within the lakes. The study also found that bacterial abundance and diversity increased with meltwater during melting seasons, and that factors like pH, conductivity, and temperature significantly influence bacterial distribution.

Remarkably, no bacterial taxon was found to be common to all habitats studied, indicating high ecosystem specificity of microbial communities. Ilahi et al*.* [75] observed a contrasting phenomenon in their samples collected from the Golen Valley in northern Pakistan. Although there were differences in ecological parameters within the study area, the differences in bacterial diversity were not significant. Their research indicated that bacterial diversity remained relatively stable in lake debris and in meltwater at the glacier margin.

Our study indicates that concentrations of different chemical elements covary with bacterial community structure (Fig. 4, 5), reflecting habitat-specific stratification (Fig. 6). The most comparable patterns were observed when ordination based on chemistry was compared with bacterial community profiles (Fig. 6, 7). Similar habitat-linked associations between glacier chemistry and bacterial assemblages have also been reported in other mountain ranges [75]. Accumulation of heavy metals in high mountain pristine glacial-fluvial environments is the result of natural weathering [76]. Rock and soil dust are the dominant sources of iron and barium and an important source of manganese [77]. Manganese variations, for which rock and soil contributions are also important, have been found to closely parallel barium variations.

Large numbers of bacteria beneath glaciers play an important role in chemical weathering and carbon cycling processes [78–80]. Abakumov et al*.* [81], who investigated a glacier in the Caucasus, found that sediments near the glacier were the main source of contamination. According to Ilahi et al*.* [75], iron, zinc, lead and copper are important driving factors in shaping microbial diversity in the glacier ecosystem. In addition, the geochemistry of bedrock and glacial soil directly influences the chemosynthetic properties of microbial communities [82]. Wu et al*.* [83], who investigated glacial catchments in the northeastern region of the Tibetan Plateau, found that, rubidium, molybdenum, barium, chromium, nickel, and other heavy elements were mainly derived from aeolian dust, riverbed sediments, and soil. Moreover, the presence of a select and narrow group of related, but not identical, microorganisms in glaciers is a consequence of the severe constraints that this environment poses to bacteria (desiccation, freezing, high pressure, and low nutrient and oxygen concentrations), which act by selecting for similar phylogenetic groups [64].

A particular trend in glacial melting today is the increased abundance of mercury [84, 85]. Our data confirm that higher mercury concentrations in glacial systems can limit bacterial growth and diversity (Fig. 5). Several studies from Tibet have shown that the levels of total mercury (THg) concentrations in glacial snow on were higher in the northern than the southern region [85–87]. Loewen et al*.* [88] suggested that particulate matter played an important role in the atmospheric transport and deposition of mercury over western China. Deposition of atmospheric mercury associated with dust particles is reported as a major factor influencing the distribution and concentration levels of mercury in glaciers [87, 88].

## Conclusion

Glacier dynamics associated with elemental concentration and their interaction with the bacterial fragment is a complex topic that remain an understudied area of research. Elements accumulate in glacial systems in multiple ways and have different effects on the microbiota in an already challenging environment. The aim of this study was to investigate this topic in the Zhongar Alatau Mountains. The glacier proved to be a reservoir of trace elements such as manganese, calcium, rubidium, potassium, barium and iron, but had low species diversity with only a few dominant species. On the other hand, glacial lake has the highest diversity and less accumulation of elements. However, in the future, the continuous melting of glaciers may lead to an increase in flow and a complete alteration of glacial cycles, leading to a change in bacterial structures and a proliferation of elements.

## Data Availability

The data are available on request from the corresponding author.
